# Co-Assembled Supramolecular Organohydrogels of Amphiphilic Zwitterion and Polyoxometalate with Controlled Microstructures

**DOI:** 10.3390/molecules29102286

**Published:** 2024-05-12

**Authors:** Peilin Wei, Yu Duan, Chen Wang, Panpan Sun, Na Sun

**Affiliations:** 1 College of Pharmacy, Shandong Second Medical University, Weifang 261053, China; 19510200661@163.com (P.W.); miaoyintian@163.com (Y.D.); wangchchw@163.com (C.W.); 2 School of Bioscience and Technology, Shandong Second Medical University, Weifang 261053, China

**Keywords:** organohydrogels, supramolecular co-assembly, zwitterionic amphiphiles, polyoxometalates

## Abstract

The organization of modifiable and functional building components into various superstructures is of great interest due to their broad applications. Supramolecular self-assembly, based on rationally designed building blocks and appropriately utilized driving forces, is a promising and widely used strategy for constructing superstructures with well-defined nanostructures and diverse morphologies across multiple length scales. In this study, two homogeneous organohydrogels with distinct appearances were constructed by simply mixing polyoxometalate (phosphomolybdic acid, HPMo) and a double-tailed zwitterionic quaternary ammonium amphiphile in a binary solvent of water and dimethyl sulfoxide (DMSO). The delicate balance between electrostatic attraction and repulsion of anionic HPMo clusters and zwitterionic structures drove them to co-assemble into homogeneous organohydrogels with diverse microstructures. Notably, the morphologies of the organohydrogels, including unilamellar vesicles, onion-like vesicles, and spherical aggregates, can be controlled by adjusting the ionic interactions between the zwitterionic amphiphiles and phosphomolybdic acid clusters. Furthermore, we observed an organohydrogel fabricated with densely stacked onion-like structures (multilamellar vesicles) consisting of more than a dozen layers at certain proportions. Additionally, the relationships between the self-assembled architectures and the intermolecular interactions among the polyoxometalate, zwitterionic amphiphile, and solvent molecules were elucidated. This study offers valuable insights into the mechanisms of polyoxometalate-zwitterionic amphiphile co-assembly, which are essential for the development of materials with specific structures and emerging functionalities.

## 1. Introduction

Molecular self-assembly represents a spontaneous natural process and a relatively common strategy for creating highly ordered nano- to micro-architectures, fostering the development of diverse functional materials with enhanced complexity, synergetic and dynamic properties. Among these materials, supramolecular gels, resulting from the self-assembly of low-molecular-weight gelators (LMWGs) through an array of supramolecular noncovalent interactions, have garnered substantial research attention due to their emerging application prospects such as biomaterials, sensing, stimulus-responsive and self-healing materials [1,2,3,4,5]. Self-assembly represents an efficient and low-energy pathway for the spontaneous generation of programmable and versatile nanostructures. By manipulating the distinct moieties, such as hydrophilicity and hydrophobicity of building blocks, along with the types of interactions between them, and controlling assembly conditions (solvent polarity, pH, light, and temperature etc.), devisable and multitudinous assemblies can be achieved, including micelles, vesicles, liquid crystals, and fibers [6,7,8,9]. Currently, the engineering of innovative and high-tech applications of conventional hydrogels has garnered significant interest. Specifically, organohydrogels, prepared in the water-organic solvent mixture, have attracted broad attention due to their intriguing properties, such as superior freezing-resistance, anti-drying capacity, and enhanced mechanical performance [10,11,12]. Therefore, there is an urgent need to develop supramolecular organohydrogels based on novel building blocks, which provide a straightforward strategy and fresh perspective for constructing diverse microstructures with additional and specific functionalities.

Among the myriad building blocks and strategies employed in constructing functional supramolecular gels, inorganic-organic co-assembly, merging the diversity of organic building blocks with the versatility of inorganic assemblies, has been considered as a promising approach. Moreover, by tailoring the specific properties of individual components, the intrinsic capabilities of resulting hybrid co-assembled systems can be precisely adjusted. Furthermore, the co-assembling process serves as a valid strategy for organizing both inorganic and organic building blocks into well-defined supramolecular nanostructures, which greatly improves properties through forming local concentrated environment, such as photochromism, luminescence, catalytic performance and antibacterial ability [13]. Various inorganic materials including polyoxometalates (POMs), quantum dots (QDs), polyhedral oligomeric silsesquioxane (POSS), have been widely employed as inorganic components to create well-defined inorganic-organic hybrid materials [14,15,16,17]. Particularly, POMs, which are anionic nanoscale inorganic clusters composed of early transition metal oxides in their highest oxidation states, exhibit diverse compositions and structures as well as versatile and tunable chemical properties, making them excellent candidates as inorganic building blocks for the construction of inorganic-organic co-assembled materials [18,19]. Due to the anionic characteristic of POMs in polar solvents, cationic amphiphiles or polymers are commonly used to co-assemble with POMs through electrostatic interactions. Wu et al. reported that nanodisks, nanocones, nanotubes, and onionlike hybrid nanostructures could be formed in co-assembling systems of POM nanoclusters and cationic amphiphiles [20,21]. Hao et al. studied that the co-assembly of double-tailed cationic surfactant possessing magnetic properties and magnetic POM clusters, which led to the formation of supramolecular magnetic aggregate structures comprised of layered structures and virus-like particles [22,23]. However, because of strong electrostatic interactions, the aforementioned co-assembled complexes are nearly insoluble in water and challenging to process into functional materials. Zwitterions, characterized by a covalently connected cation and anion, can interact with various ionic species and co-assemble into diverse aggregates via electrostatic forces. Recently, the delicate balance between electrostatic attraction and repulsion of zwitterionic amphiphiles and POM clusters has been discovered, leading to the successful construction of homogenous supramolecular hydrogel systems. In our previous studies, single-tailed imidazolium-type zwitterions were commonly employed to co-assemble with POM clusters, resulting in the observation that these systems exhibited a distinct tendency to form well-organized wormlike micelles [24,25]. Consequently, appropriately designing the structure of building blocks to regulate driving forces represents a rational strategy for generating diverse assemblies and investigating the assembly mechanism.

In this work, we designed and synthesized a double-tailed zwitterionic quaternary ammonium amphiphile (dioctylmethylammonium propanesulfonate, (C8)_2_MeAS) for co-assembly with Keggin-type POM clusters (phosphomolybdic acid, HPMo), followed by a systematic investigation of their co-assembled characteristics. Leveraging the suitable ionic interaction between zwitterionic amphiphiles and anionic POM clusters, (C8)_2_MeAS and HPMo clusters were able to co-assemble into supramolecular organohydrogels with tunable nanostructures in a binary solvent of water and dimethyl sulfoxide (DMSO) (4:1 *v*/*v*). The morphologies of co-assemblies inside the supramolecular organohydrogels transformed from onion-like vesicles to spherical aggregates as the molar ratio of (C8)_2_MeAS to HPMo varied from 10:1 to 6:1. The gelation behavior, gelation mechanism, and rheological properties were detailed studied to reveal a theoretical comprehension of zwitterionic amphiphiles-POMs co-assembly in the binary solvent. Zwitterionic amphiphiles significantly broaden the scope of POM clusters’ applications as building blocks in the fabrication of versatile co-assembled nanostructures and soft materials.

## 2. Results and Discussion

### 2.1. Phase Diagram and Morphologies of the Organohydrogels

**Figure 1:**
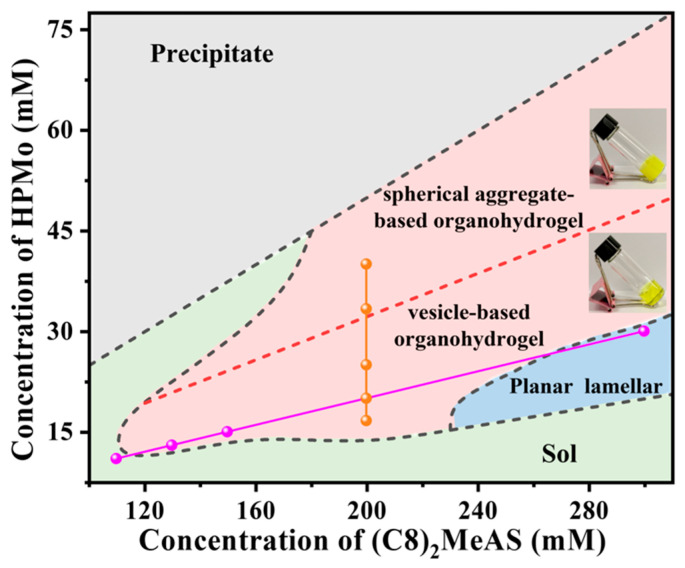
Phase diagram of mixtures of (C8)_2_MeAS and HPMo at different molar ratios in water-DMSO binary solvent (4:1 *v*/*v*); the orange points represent samples with a fixed (C8)_2_MeAS concentration of 200 mM; the pink points represent samples with a fixed (C8)_2_MeAS/HPMo molar ratio of 10:1; the insets are photos of organohydrogels with the molar ratios of 10:1 and 6:1, respectively (at a fixed (C8)_2_MeAS concentration of 200 mM).

The zwitterionic compound (C8)_2_MeAS exhibits limited solubility in water because of its two hydrophobic alkyl chains. To enhance the solubility of (C8)_2_MeAS and induce more complex co-assembly behavior, DMSO was used as a cosolvent [26,27]. To investigate the co-assembling behavior of zwitterionic (C8)_2_MeAS and anionic HPMo clusters in a water-DMSO binary solvent, the phase diagram of (C8)_2_MeAS/HPMo system at various (C8)_2_MeAS concentrations and varying (C8)_2_MeAS/HPMo molar ratios in water-DMSO binary solvent (4:1 *v*/*v*) was primarily examined. As illustrated in Figure 1, the phase boundary was primarily determined through visual observation and the tube-inversion method. (C8)_2_MeAS cannot dissolve on its own in a mixture of water-DMSO mixed solvent with the volume ratio of 4:1. The addition of hydrophilic HPMo clusters significantly increases the solubility of (C8)_2_MeAS, resulting in the formation of a homogenous solution. With the successive increase of HPMo concentration, planar lamellar phase (L_α_), gel phase and precipitate region was observed. The insets of Figure 1 demonstrate that gels with (C8)_2_MeAS/HPMo molar ratio from 7:1 to 12:1 exhibit good transparency, while gels with (C8)_2_MeAS/HPMo molar ratio of 5:1 and 6:1 are optically opaque. For a more detailed examination of gel phase, representative samples with a fixed (C8)_2_MeAS concentration of 200 mM were initially chosen for specific analysis (depicted by orange points in Figure 1). Optical photographs of organohydrogels, observed under crossed polarizers, reveal a birefringent texture in samples with (C8)_2_MeAS/HPMo molar ratios of 12:1, 10:1 and 8:1 (Figure S1a). Additionally, the phase transition of the samples at a constant (C8)_2_MeAS/HPMo molar ratio of 10:1 was investigated (indicated by the pink points in Figure 1). When the concentration of (C8)_2_MeAS is lower than 120 mM, a homogeneous and transparent solution with no birefringence was obtained (Figure S1b). Optical photographs of organohydrogels with (C8)2MeAS concentration of 130 mM and 150 mM observed under crossed polarizers show birefringent textures (Figure S1b). This suggests that a transition from a micellar solution to a vesicle-based gel phase occurs at a (C8)_2_MeAS concentration of 130 mM. Upon increasing the concentration of (C8)_2_MeAS to 270 mM, the optically transparent gel sample turned to a viscous, translucent, and flowing substance exhibiting an oil-like texture under polarized optical microscope (POM) (Figure S3). These results demonstrate that a phase transition from vesicles to planar lamellar structures occurs as the concentration of (C8)_2_MeAS increases to higher values.

**Figure 2:**
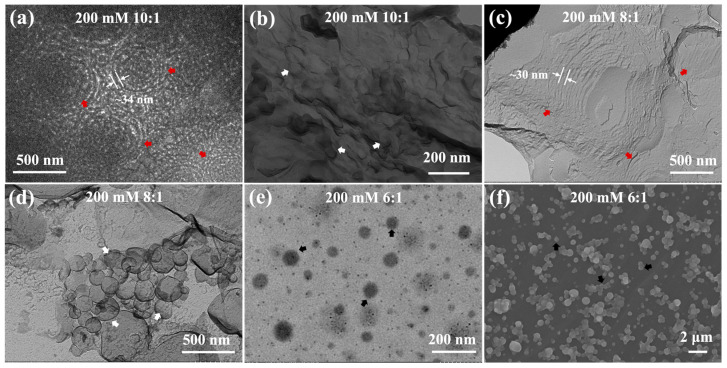
(**a**,**b**) Cryo-TEM and FF-TEM images of organohydrogel with the (C8)_2_MeAS/HPMo molar ratio of 10:1; (**c**,**d**) FF-TEM and cryo-TEM images of organohydrogel with the (C8)_2_MeAS/HPMo molar ratio of 8:1; (**e**,**f**) TEM and SEM images of organohydrogel with the (C8)_2_MeAS/HPMo molar ratio of 6:1. The concentration of (C8)_2_MeAS was fixed at 200 mM. The unilamellar vesicles, multilamellar vesicles and spherical aggregates in the images have been depicted by white, red and black arrows, respectively.

**Figure 3:**
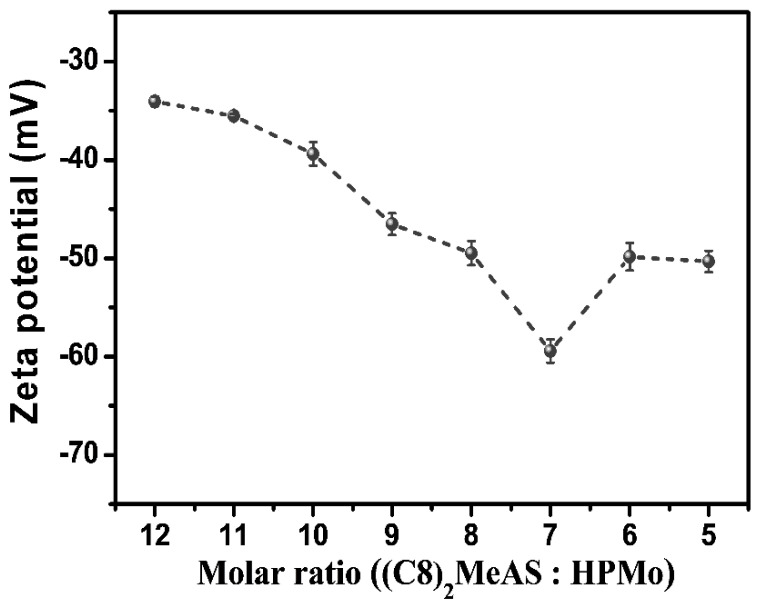
Zeta potentials for mixtures at (C8)_2_MeAS concentration of 90 mM with different (C8)_2_MeAS/ HPMo molar ratios.

To further visualize and identify the inner microstructure of assembled organohydrogels, cryogenic transmission electron microscopy (cryo-TEM) and freeze fracture-transmission electron microscope (FF-TEM) were conducted. Figure 2a,b display the cryo-TEM and FF-TEM images of the organohydrogel with a (C8)_2_MeAS/HPMo molar ratio of 10:1. Densely stacked onion-like vesicles (multilamellar vesicles) and accompanying unilamellar vesicles are clearly observed. The onion-like vesicles have diameters ranging from 380 to 700 nm and interlamellar distances of approximately 34 nm. The unilamellar vesicles have smaller diameters ranging from 54 to 110 nm. As shown in Figure 2c,d, the organohydrogel with a (C8)_2_MeAS/HPMo molar ratio of 8:1 also consists of large multilamellar vesicles with the diameters ranging from 850 nm to 1.4 μm, along with polydispersed unilamellar vesicles with diameters ranging from 50 to 500 nm. Multilamellar vesicles exhibit a smaller bilayer distance of about 30 nm compared to those in the organohydrogel of a 10:1 ratio. Moreover, slight agglomeration of unilamellar vesicles occurs in both organohydrogels with 10:1 and 8:1 ratios, promoting gel formation [28,29]. Increasing the (C8)_2_MeAS/HPMo molar ratio to 6:1 resulted in a morphological transformation from vesicles to spherical aggregates. As shown in Figure 2e,f, the organohydrogel with a 6:1 ratio consists of polydisperse and well-defined spherical aggregates ranging in diameter from about 30 nm to near 1 μm, corresponding to the opaque appearance of organohydrogels [30]. Scanning electron microscopy (SEM) images reveal the adhesion of spherical aggregates, leading to network formation and subsequent gelation [31]. Similar spherical aggregates can be observed for the organohydrogel with a 5:1 ratio (Figure S2). As mentioned earlier, the morphological transformation with the increase in HPMo content is further confirmed by zeta potential measurements, typically used to monitor the surface potential of aggregates in colloidal systems. Figure 3 shows the zeta potentials of mixtures with a constant (C8)_2_MeAS concentration and varying (C8)_2_MeAS/HPMo molar ratios. The negative zeta potential of the mixtures demonstrates that anionic POM clusters reside the surface of the aggregates [17]. The zeta potential becomes increasingly negative as the (C8)_2_MeAS/HPMo molar ratio varies from 12:1 and 7:1. The interaction between POM clusters and the covalently connected cation and anion of zwitterionic amphiphiles via electrostatic forces results in the bilayers becoming more negatively charged upon incorporation of more anionic POM clusters [24,32]. However, the zeta potential becomes less negative as the HPMo content increases to molar ratios of 6:1 and 5:1, corresponding with the observed morphological transformation. The slight increase in zeta potential might be attributed to the accumulation of counterions (H^+^) on the aggregate surface upon addition of excess HPMo [33].

**Figure 4:**
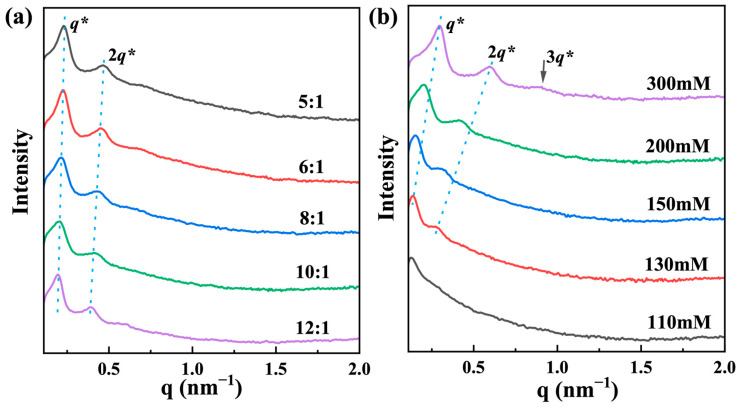
(**a**) SAXS spectra of samples with different (C8)_2_MeAS/HPMo molar ratios and fixed (C8)_2_MeAS concentration of 200 mM; (**b**) SAXS data of samples with various (C8)_2_MeAS concentrations and the fixed (C8)_2_MeAS/HPMo molar ratio of 10:1.

Small-angle X-ray scattering (SAXS) measurement is a potent technique for examining co-assembled microstructures in samples. Firstly, SAXS spectra were detected for samples with different molar ratios of (C8)_2_MeAS/HPMo and fixed (C8)_2_MeAS concentration of 200 mM (Figure 4a). Two distinctive scattering peaks, corresponding to the (100) and (200) reflections of lamellar structures, were observed with a relative ratio of 1:2. This observation concurs with the presence of multilamellar vesicles as depicted in TEM images. The interlayer spacing (*d*) can be determined using the equation *d* = 2*π*/*q*, derived from the first scattering factor (*q*_1_). As the HPMo content increases from 12:1 to 5:1, the scattering peaks gradually shift towards higher *q* values, indicating a reduction in interlayer spacing from 31.08 to 26.04 nm. This trend suggests a denser packing of bilayers [28]. The introduction of HPMo molecules on the bilayer surface enhances interactions between adjacent bilayers, resulting in a more tightly packed bilayer structure. For organohydrogels with the (C8)_2_MeAS/HPMo molar ratios of 10:1 and 8:1, the calculated *d*-spacing is 31.08 and 28.34 nm, respectively. Then, the SAXS patterns were measured for samples with a constant (C8)_2_MeAS/HPMo molar ratio of 10:1 and varying (C8)_2_MeAS concentrations, as shown in Figure 4b. In the case of the sample with a (C8)_2_MeAS concentration of 110 mM, no significant scattering peak is observed. However, with an increase in (C8)_2_MeAS concentration to 130 mM, scattering peaks corresponding to lamellar structures emerge. Notably, these scattering peaks become sharper with increasing (C8)_2_MeAS concentration, indicating the more regular arrangement of bilayers in the samples. Increasing the (C8)_2_MeAS concentration to 300 mM results in evident peaks at *q* values in a 1:2:3 ratio, indicating more ordered planar lamellar structures. With the (C8)_2_MeAS concentration rising from 130 mM to 300 mM, the *d*-spacing reduces from 43.80 nm to 21.41 nm. This can be attributed to the more closely packed molecules with increasing (C8)_2_MeAS concentration [28,34]. Thus, the augmentation of both HPMo and (C8)_2_MeAS concentration ineluctably leads to a reduction in interlayer spacing of bilayers.

### 2.2. Rheological Properties of the Organohydrogels

**Figure 5:**
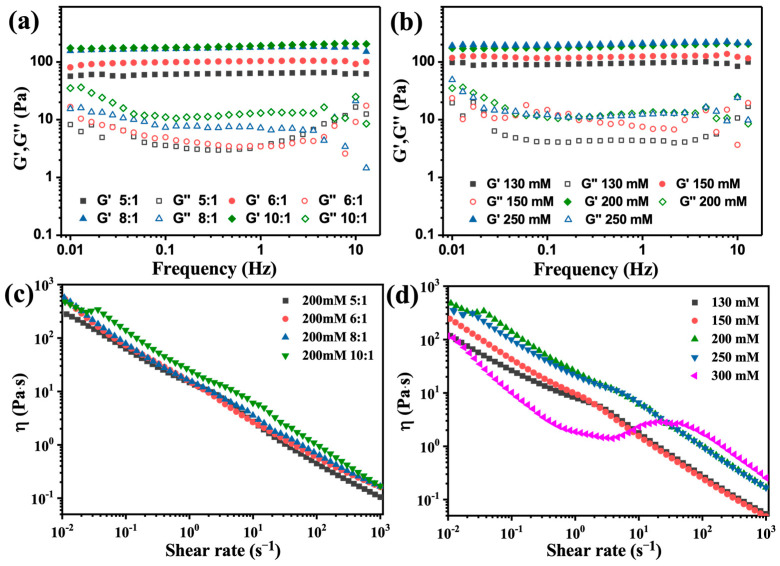
The rheological properties of the prepared samples: (**a**,**c**) frequency-dependent oscillatory profiles and shear viscosity as a function of shear rate for organohydrogels with different (C8)_2_MeAS/HPMo molar ratios (The concentration of (C8)_2_MeAS was fixed at 200 mM); (**b**,**d**) frequency sweep plots and shear rate dependence of the viscosity for organohydrogels with varying (C8)_2_MeAS concentration ((C8)_2_MeAS/HPMo = 10:1).

Rheological measurements are generally used to investigate the macroscopic properties of samples. Figure 5a shows frequency sweep results for organohydrogels with different (C8)_2_MeAS/HPMo molar ratios. The elastic moduli (G′) of all samples exceed the viscous moduli (G″) by approximately one order of magnitude. Furthermore, the G′ and G″ curves run almost parallel and remain frequency-independent, further certifying the good gel character of samples [35]. With the (C8)_2_MeAS/HPMo molar ratio ranging from 10:1 to 5:1, the G′ value, a significant parameter for assessing gel mechanical strength, gradually decreases, implying reduced ability to resist mechanical disturbance [24]. The decrease of mechanical strength arises from two factors: the microstructures within the gels and excessive crosslinking by HPMo clusters. Obviously, the G′ values of vesicle-based organohydrogels (8:1 and 10:1) higher than those of spherical aggregate-based organohydrogels (5:1 and 6:1). Vesicle-based gels feature densely packed multilamellar and unilamellar vesicles with minimal inter-vesicle spacing, forming a notably stiffer network compared to gels composed of adhesive spherical aggregates. Moreover, because of the hydrophilicity of POMs clusters, HPMo clusters situated on the outer surface of adjacent aggregates can interact with each other and serve as cross-linkers, facilitating the binding of neighboring aggregates and promoting gel formation [24]. However, an excessive addition of HPMo clusters may result in an increase in electrostatic repulsive forces among neighboring aggregates, significantly weakening the crosslinking of HPMo clusters. In addition, we also explored the frequency-dependent oscillatory profiles of organohydrogels with different (C8)_2_MeAS concentrations but a fixed (C8)_2_MeAS/HPMo molar ratio of 10:1 (Figure 5b). Both G′ and G″ remain independent of frequency. Moreover, all samples possess higher G′ values, indicating their elasticity dominant property, a characteristic feature of gel materials. The mechanical strength of organohydrogels gradually enhances with the rise in (C8)_2_MeAS content. Summarily, the mechanical strength of gels can be facilely tuned by altering the content of (C8)_2_MeAS and HPMo clusters.

Additionally, the microstructure transformation of self-assembled systems could be distinguished from rheological measurements. Steady-shear measurements for samples with various components were further conducted. In Figure 5c, the viscosity of organohydrogels, with a fixed (C8)_2_MeAS concentration of 200 mM, decreased gradually as the shear rate increased, implying the good shear-thinning property of gels. Therefore, these organohydrogels have potentially application as injectable materials. In Figure 5d, for organohydrogels with fixed (C8)_2_MeAS/HPMo molar ratios of 10:1, the viscosity of gels rises with increasing (C8)_2_MeAS concentration. This occurs because the increasing number of molecules results in tighter packing of vesicles within the organohydrogel. At a (C8)_2_MeAS content of 300 mM, the sample transitioned into a viscous fluid with a planar lamellar structure, resulting in a significant decreased in viscosity. Notably, a partial shear thickening behavior is observed within the shear rate range of 4 s^−1^ to 47 s^−1^, potentially associated with the transition from planar bilayers to onion-like vesicles. This transition from lamellar to onion-like structures is a universal phenomenon in certain surfactant/water system [36,37,38]. The subsequent shear-thinning region is attributed to the deformation of vesicle bilayers as the shear rate increases.

### 2.3. Co-Assembly Mechanism of the Formation of Organohydrogels

During the co-assembly process of the (C8)_2_MeAS/HPMo system, an essential prerequisite is the matching of hydrophobicity of the zwitterionic amphiphiles with the hydrophilicity of the POMs. When the HPMo clusters co-assembled with either significantly more hydrophilic dihexylmethylammonium propanesulfonate ((C6)_2_MeAS) or notably more hydrophobic didecylmethylammonium propanesulfonate ((C10)_2_MeAS), dilute aqueous solution and precipitates were obtained at equivalent concentrations, respectively (Figure S4).

**Figure 6:**
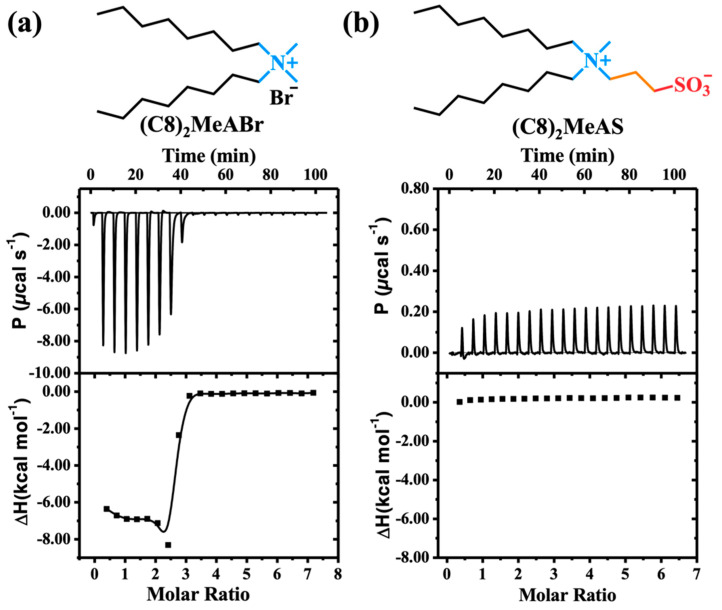
ITC titration data of (**a**) ammonium-type cationic amphiphile ((C8)_2_MeABr, 3 mM) and (**b**) zwitterionic amphiphile ((C8)_2_MeAS, 3 mM) titrated into HPMo solution (0.1 mM) at 25.0 °C. The water-DMSO binary solvent with the volume ration of 4:1 was used to prepare above solutions.

Another crucial factor contributing to the abundant phase behavior is the delicate interaction between zwitterions and POM clusters. Isothermal titration calorimetry (ITC) technique was employed to explore the thermodynamic behavior in the amphiphile-POMs mixing process. In the case of the cationic amphiphile ((C8)_2_MeABr) and the anionic HPMo system (Figure 6a), an exothermic process with a distinct enthalpy change occurred during titration, confirming the strong electrostatic attraction between (C8)_2_MeABr and HPMo clusters. This strong electrostatic attraction between the cationic (C8)_2_MeABr and the anionic HPMo induces the formation of light-yellow precipitation (Figure S4). Conversely, as depicted in Figure 6b, enthalpy value of the endothermic process is low and remains relatively constant during the binding process of zwitterionic (C8)_2_MeAs and HPMo, resulting from the covalently connected cation and anion of the zwitterion. For zwitterionic (C8)_2_MeAS, along with the electrostatic attraction between the positively charged quaternary ammonium cation and the [PMo_12_O_40_]^3−^ anion, the electrostatic repulsion also exists between the sulfonic anion and the [PMo_12_O_40_]^3−^ anion, which is crucial for maintaining the electrostatic balance, as shown in Figure S5. The appropriate balance between electrostatic attraction and repulsion results in small enthalpy values in the ITC measurement and facilitates the formation of diverse phase behaviors and homogenous gel systems.

**Figure 7:**
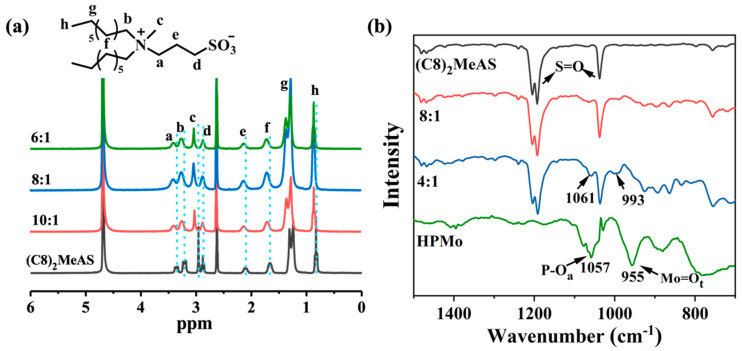
(**a**) ^1^H NMR of mixtures of (C8)_2_MeAS and HPMo in D_2_O/DMSO-*d*_6_ with various (C8)_2_MeAS/HPMo molar ratios (the concentration of (C8)_2_MeAS is fixed at 5 mM, the volume ratio of D_2_O/DMSO-*d*_6_ is 4:1); (**b**) FT-IR spectra of (C8)_2_MeAS, HPMo and xerogels with the (C8)_2_MeAS/HPMo molar ratios of 4:1 and 8:1.

The intermolecular noncovalent forces involved in the co-assembly process were further investigated. The interaction between zwitterionic amphiphiles and HPMo clusters during co-assembly was initially studied using ^1^H NMR spectra (Figure 7a). Protons adjacent to the charge (H_a_~H_d_) of (C8)_2_MeAS molecules in non-co-assembled state (the black line) exhibit much sharper peaks compared to the protons of (C8)_2_MeAS molecules in the co-assembled state. Furthermore, the protons of (C8)_2_MeAS exhibit a noticeable downfield shift after mixing with the HPMo cluster, ascribing to the altered dielectric constant of the solvent and the conformational effects induced by molecular aggregation in the presence of HPMo [39,40]. Moreover, with the continuous increase in HPMo content, the proton of the methyl on the quaternary ammonium cation further shifts slightly downfield, attributed to the electrostatic interaction between (C8)_2_MeAS and HPMo clusters. Fourier transform infrared spectroscopy (FT-IR) was also carried out to further elucidate the interaction mechanism between (C8)_2_MeAS and HPMo clusters. FT-IR spectra of (C8)_2_MeAS, HPMo, and xerogels with different (C8)_2_MeAS/ HPMo molar ratios are shown in Figure 7b. The fundamental absorptions of HPMo clusters, including the asymmetric P−O_a_ stretch at 1057 cm^−1^ and the Mo=O terminal stretch (Mo=O_t_) at 955 cm^−1^, are observed [41]. After co-assembling with (C8)_2_MeAS, the vibrational characteristics of HPMo are still preserved. In comparison with the pure HPMo clusters, the absorptions of P−O_a_ and Mo=O_t_ shift to 1061 cm^−1^ and 993 cm^−1^ respectively, which can be ascribed to the intermolecular electrostatic interactions between HPMo and (C8)_2_MeAS [24]. The results of ^1^H NMR and FT-IR experiments above reveal that intermolecular interaction plays an essential role in the co-assembly process.

**Scheme 1:**
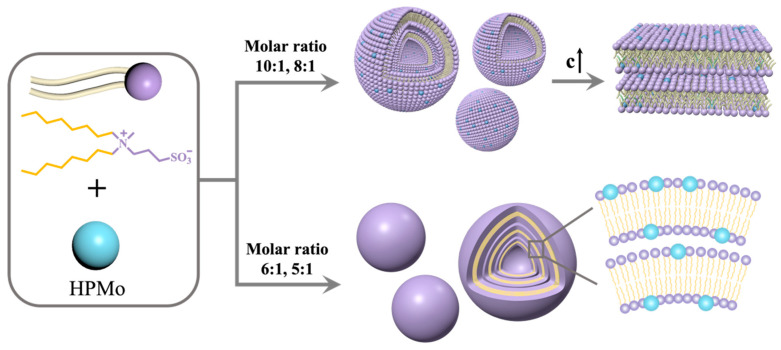
The proposed mechanism for the formation of different organohydrogels.

The results above unequivocally demonstrate the critical role of electrostatic interaction between (C8)_2_MeAS molecules and HPMo in the formation of organohydrogels with diverse microstructures. For the case of molar ratio at 10:1 and 8:1, HPMo cluster primarily increase the solubility of zwitterionic amphiphiles. Consequently, the morphology of co-assemblies mainly dictated by the geometric properties of (C8)_2_MeAS amphiphile. The topological structure of (C8)_2_MeAS is similar to the conventional double-tailed amphiphiles, which prefers to form bilayers in solution [23,42]. In the solvent system of water-DMSO, the charged zwitterionic headgroups reside outside of the vesicles, while hydrophilic HPMo clusters anchor at the interface through electrostatic interactions. Furthermore, HPMo clusters located on the outer surface of vesicles can interact with HPMo clusters on adjacent vesicles by virtue of hydrogen-bonding, van der Waals forces and electrostatic interactions. These interactions lead to the adhesion and rearrangement of bilayers, facilitating the formation of multilamellar vesicles. With the increasing concentrations of (C8)_2_MeAS and HPMo, a sturdy network of multilamellar and unilamellar vesicles is formed, retaining the solvent and promoting gel formation. The probably mechanism for the transformation of aggregates is proposed, as shown in Scheme 1. As the molar ratio changes to 6:1 and 5:1, the transition in morphology can be explained by the surface occupation model [18,20]. At these molar ratios, the occupancy of zwitterionic amphiphiles on the POM core is less than 1/3, favoring a linear-shaped geometry wherein (C8)_2_MeAS resides predominantly on one side of POM core, thus, promoting bilayer formation. On account of the excessive interactions between HPMo clusters on adjacent bilayers, the bilayer structure transformed into well-defined spherical assemblies with tidily and closely packed bilayers. Such HPMo cluster-cluster interactions among adjacent spherical particles lead to a network of these spherical aggregates, consequently resulting in the formation of organohydrogels [43].

**Figure 8:**
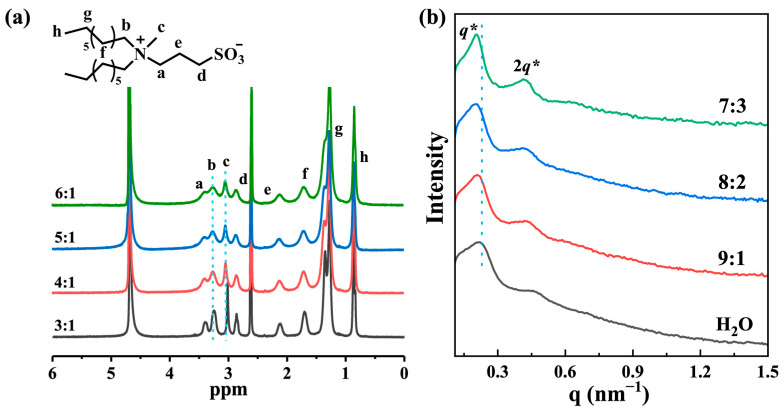
(**a**) ^1^H NMR spectra of mixtures of (C8)_2_MeAS and HPMo in the binary solvent with different D_2_O/DMSO-*d*_6_ volume ratios; (**b**) SAXS spectra of samples prepared in water-DMSO mixed solvent with various volume ratios (the concentration of (C8)_2_MeAS is fixed at 200 mM; (C8)_2_MeAS/HPMo = 10:1).

The aggregation behavior of amphiphilic molecules usually can be manipulated by the presence of a cosolvent [27,29]. Thus, we explored the effect of DMSO content on the co-assembly process of (C8)_2_MeAS and HPMo. ^1^H NMR spectra of mixtures of (C8)_2_MeAS and HPMo in a binary solvent with different D_2_O/DMSO-*d*_6_ volume ratios were recorded, as shown in Figure 8a. Despite varying the D_2_O/DMSO-*d*_6_ volume ratio from 3:1 to 6:1, the protons located on (C8)_2_MeAS did not show obvious shift, indicating that the altered polarity of the binary solvent with increasing DMSO-*d*_6_ volume has minimal impact on the electrostatic interaction between (C8)_2_MeAS and HPMo [44]. SAXS spectra of samples prepared in water-DMSO mixed solvent with various volume ratios were also measured (Figure 8b). All SAXS spectra exhibit typical scatting peaks corresponding to lamellar structures with a *q* value ratio of 1:2. Relative to samples prepared in pure water, those in a water-DMSO mixed solvent (9:1) exhibit an increase in *d*-spacing from 28.38 nm to 31.08 nm, suggesting the looser bilayer structure in the presence of DMSO. The amphiphilic (C8)_2_MeAS molecule is completely soluble in pure DMSO. Some of the added DMSO molecules solubilize into the hydrophobic layer of aggregates, causing the swelling of bilayers, and eventually resulting in an increase in *d*-spacing [45,46,47]. Further augmenting the water-DMSO ratio from 9:1 to 7:3, there is no significant variation of d-spacing, revealing that alterations in solution polarity do not affect the internal morphology. Noticeably, with the increasing DMSO content, the peaks become much sharper, indicating the lamellar structure turns to more ordered.

## 3. Materials and Methods

### 3.1. Materials

The compounds N-methyl-N-octyl-1-octanamine (98%), 1, 3-propanesultone (99%) and dimethyldioctylammonium bromide ((C8)_2_MeBr, 98%) were purchased from J&K Scientific Co., Ltd. (Beijin, China). Phosphomolybdic acid (HPMo, 99%) was acquired from Aladdin Co., Ltd. (Shanghai, China). Dimethyl sulfoxide (DMSO) was obtained from Sinopharm Chemical Reagent Co., Ltd. (Shanghai, China). All materials were used as received. Deionized water was used throughout all experiments.

### 3.2. Synthesis of Dioctylmethylammonium Propanesulfonate ((C8)_2_MeAS)

(C8)_2_MeAS was synthesized according to the literature method [48]. More specifically, to a solution of N-methyl-N-octyl-1-octanamine in acetonitrile placed in a round bottom flask, the equimolar amount of 1,3-propanesultone was slowly added under a nitrogen atmosphere. The resulting mixture was refluxed at 80 °C for 5 days. After the solvent was removed by filtration, the residue was washed at least three times with diethyl ether and ethyl acetate respectively. Then, the white podwer was dried in vacuum oven at room temperature for 24 h. The ^1^H NMR spectrum of (C8)_2_MeAS in CDCl_3_ is depicted in Figure S6. ^1^H NMR (400 MHz, CDCl_3_, δ/ppm): 3.716–3.770 (2H, m), 3.217–3.248 (4H, m), 3.151 (3H, s), 2.907–2.950 (2H, t), 2.209–2.260 (2H, m), 1.689 (4H, s), 1.274–1.348 (20H, m), 0.864–0.908 (6H, t).

### 3.3. Sample Preparation

Specific molar concentrations of (C8)_2_MeAS and HPMo were weighted into stoppered glass vials. Water-DMSO mixture with volume ratio of 4:1 was served as solvent. The samples were dissolved and homogenized at 60 °C, and then, equilibrated at 25 °C for at least two weeks.

### 3.4. Characterization

^1^H NMR Measurements. ^1^H NMR spectroscopy was measured on a Bruker Avance II 400 MHz NMR spectrometer equipped with a pulse field gradient module (Z-axis) using a 5 mm BBO probe at 25 °C. 

ITC Measurements. ITC were performed using an MicroCal VP-ITC apparatus at 25 °C. The 3 mM water-DMSO solution of (C8)_2_MeAS and (C8)_2_MeBr were titrated into a 0.1 mM water-DMSO solution of HPMo (1.4 mL) by using a 300 μL syringe, respectively. The total injection was 29 drops.

FT-IR Measurements. FT-IR spectra of (C8)_2_MeAS, HPMo and freeze-dried hydrogels were measured by using an FT-IR spectrometer (PerkinElmer Spectrum Two) in the range 4000–450 cm^−1^. 

Cryo-TEM and FF-TEM Observations. The preparation of samples for testing followed established methods in the literature [42,49]. For cryo-TEM, samples were prepared using a controlled environment vitrification system (CEVS) maintained at 25 °C with 95% relative humidity. A micropipette was utilized to apply 5 μL of organohydrogels onto a TEM copper grid, which was subsequently blotted with two pieces of filter paper, resulting in the formation of thin films suspended across the mesh holes. After approximately 10 s, the samples were swiftly plunged into a reservoir of liquid ethane (cooled by nitrogen) at −165 °C. The vitrified samples were preserved in liquid nitrogen until transferred to a cryogenic sample holder (Gatan 626). The preparation procedure for FF-TEM is described below. A small amount of the organohydrogel was placed on a 0.1 mm thick copper disc, covered with a second copper disc, and then, plunged into liquid propane cooled by liquid nitrogen. Fracturing and replication were conducted using Balzers BAF-400D equipment at −150 °C. Pt/C was deposited at an angle of 45°. Cryo-TEM and FF-TEM images of the organohydrogels were acquired using a transmission electron microscope (JEOL JEM-1400, 120 kV).

TEM and SEM Observations. The sample preparation involved loading about 5 μL of organohydrogels onto a carbon-coated copper grid and removing the excess organohydrogels with filter paper after 5 min. Subsequently, samples were rapidly dehydrated via freeze-drying. TEM (JEOL JEM-1400, 120 kV) was used to characterize the microcosmic structures. SEM images were observed by scanning electron microscopy (SEM, JEOL JSM-7600F).

Zeta potential Measurements. Zeta potentials were measured at 25 °C by using a Brookhaven ZetaPALS instrument. The concentration of (C8)_2_MeAS in the solutions was fixed at 100 mm. The molar ratio of (C8)_2_MeAS /HPMo was varied from 5:1 to 10:1. 

SAXS Measurements. The spectra of SAXS were recorded on the SAXSess mc2 X-ray scattering system (Anton Paar) with Cu Κ_α_ radiation (0.154 nm) operating at 50 kV and 40 mA. The distance between the sample and detector was about 264.5 mm and the wavelength of X-rays was 1.542 Å. The exposure time was 600 s for all samples.

Rheological Measurements. The rheological measurements were conducted by using a ThermoHaake RS300 rheometer with a parallel plate on a Peltier plate at 25 °C. Dynamic stress sweep spectra were recorded at a frequency of 1 Hz. Dynamic frequency sweep was carried out in the linear viscoelastic region determined from dynamic stress sweep measurements. The steady-state shear measurements were conducted with the shear rate increasing from 0.01 to 1000 s^−1^.

## 4. Conclusions

In summary, we have successfully fabricated a series of supramolecular organohydrogels through the co-assembly of double-tailed zwitterionic quaternary ammonium amphiphiles and POMs in a water-DMSO binary solvent. The organohydrogels, featuring unilamellar vesicles, onion-like vesicles, and spherical aggregates with a layered structure, were formed by controlling the ionic interaction between zwitterionic amphiphiles and HPMo clusters. The addition of DMSO barely affected the electrostatic interaction between zwitterionic amphiphiles and POM clusters. Notably, DMSO molecules are able to increase the solubility of the hydrophobic layer of aggregates and induce bilayer swelling, resulting in a more relaxed bilayer structure. The inspiration drawn from the combination of zwitterionic amphiphiles and POMs in this work provides a vivid strategy and fresh perspective for designing functional hybrid materials incorporating nanoclusters or nanoparticles with rich potentials in addition to POMs.

## Data Availability

Data are contained within the article and Supplementary Materials.

## Supplementary Materials

The following supporting information can be downloaded at: https://www.mdpi.com/article/10.3390/molecules29102286/s1, Figure S1: Optical photographs of organohydrogels observed with crossed polarizers; Figure S2: TEM and SEM images of the organohydrogel with (C8)_2_MeAS/HPMo molar ratio of 5:1; Figure S3: POM images of samples with different (C8)_2_MeAS concentration of 280 mM and 300 mM; Figure S4: Photos of mixtures composed of different amphiphiles and HPMo; Figure S5: Schematic illustration of electrostatic interaction for (C8)_2_MeABr/HPMo and (C8)_2_MeAS/HPMo systems.
