# Supplementary material for: Co-Assembled Supramolecular Organohydrogels of Amphiphilic Zwitterion and Polyoxometalate with Controlled Microstructures
Source: Molecules. 2024 May 12;29(10):2286. doi: 10.3390/molecules29102286 (PMC11124011; doi:10.3390/molecules29102286)
Supplement: Supplementary file 1 [file molecules-29-02286-s001.zip › molecules-3006002-supplementary.pdf]

Suppporting Information for

Co-assembled Supramolecular Organohydrogels of Amphiphilic Zwitterion and Polyoxometalate with Controlled Microstructures

Peilin Wei [1,†], Yu Duan [1,†], Chen Wang [1], Panpan Sun [2,*] and Na Sun [1,*]

1 College of Pharmacy, Shandong Second Medical University, Weifang, Shandong, 261053, China; 19510200661@163.com; miaoyintian@163.com; wangchchw@163.com

2 School of Bioscience and Technology, Shandong Second Medical University, Weifang, Shandong, 261053, China; sunpanpan@sdsmu.edu.cn

† These authors contributed equally to this work.

* Correspondence: sunpanpan@sdsmu.edu.cn; sunna@sdsmu.edu.cn

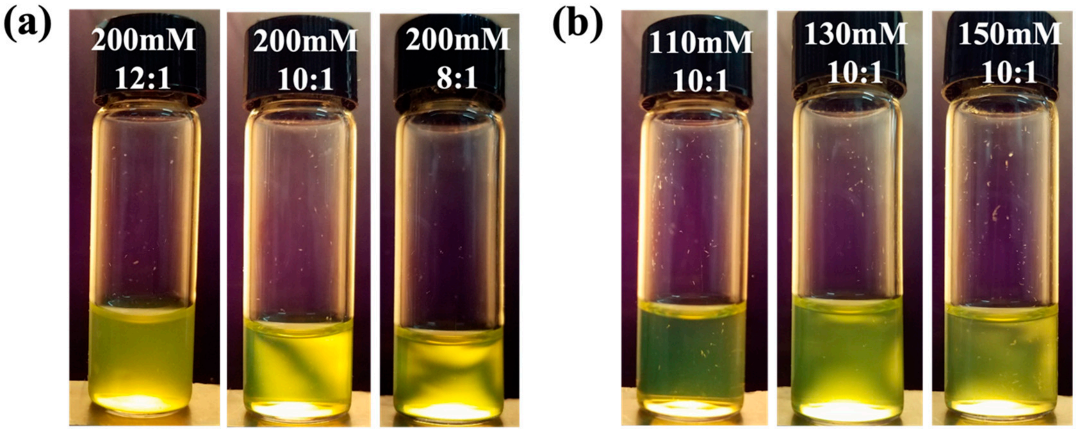

Figure S1. Optical photographs of organohydrogels observed with crossed polarizers: (a) samples with different $(C8)_2$MeAS/HPMo molar ratios and fixed $(C8)_2$MeAS concentration of 200 mM; (b) samples with various $(C8)_2$MeAS concentrations and the fixed $(C8)_2$MeAS/HPMo molar ratio of 10:1.

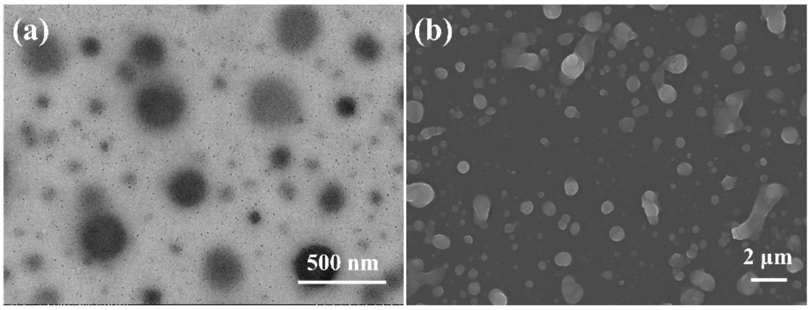

Figure S2. TEM (a) and SEM (b) images of the organohydrogel with $(C8)_2$MeAS/HPMo molar ratio of 5:1. The concentration of $(C8)_2$MeAS was fixed at 200 mM.

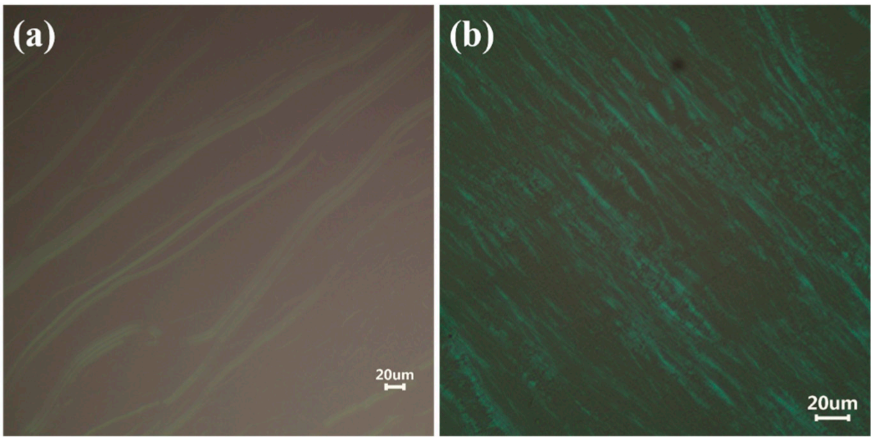

Figure S3. POM images of samples with different $(C8)_2$MeAS concentration: (a) 280 mM (b) 300 mM. The $(C8)_2$MeAS/HPMo molar ratio is fixed at 10:1.

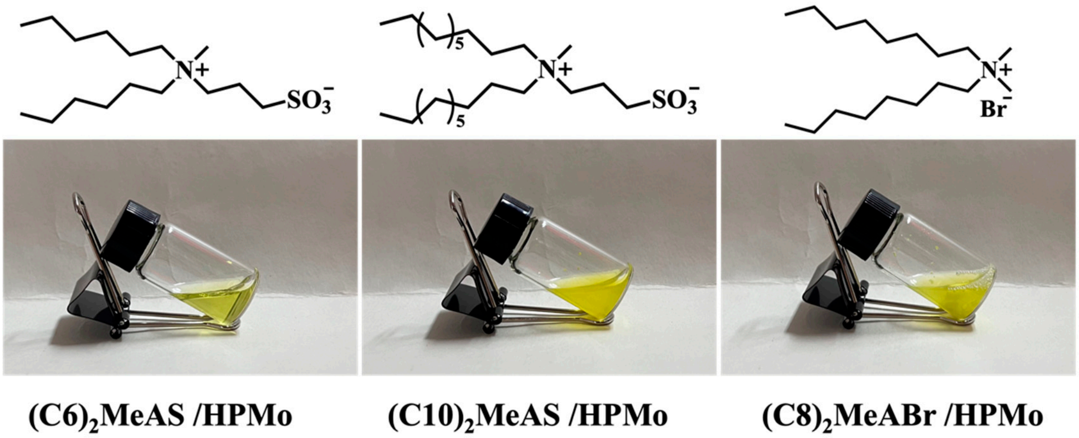

Figure S4. Photos of mixtures composed of different amphiphiles and HPMo. The molar ratio between amphiphiles and HPMo was 10:1, and the concentration of amphiphiles was fixed at 200 mM.

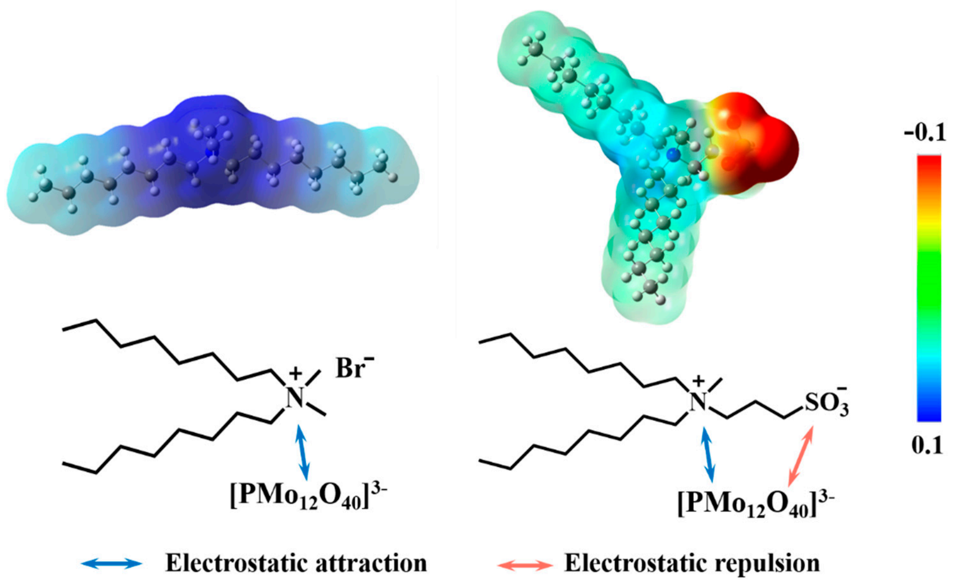

Figure S5. Schematic illustration of electrostatic interaction for $(C8)_2MeABr/HPMo$ and $(C8)_2MeAS/HPMo$ systems.

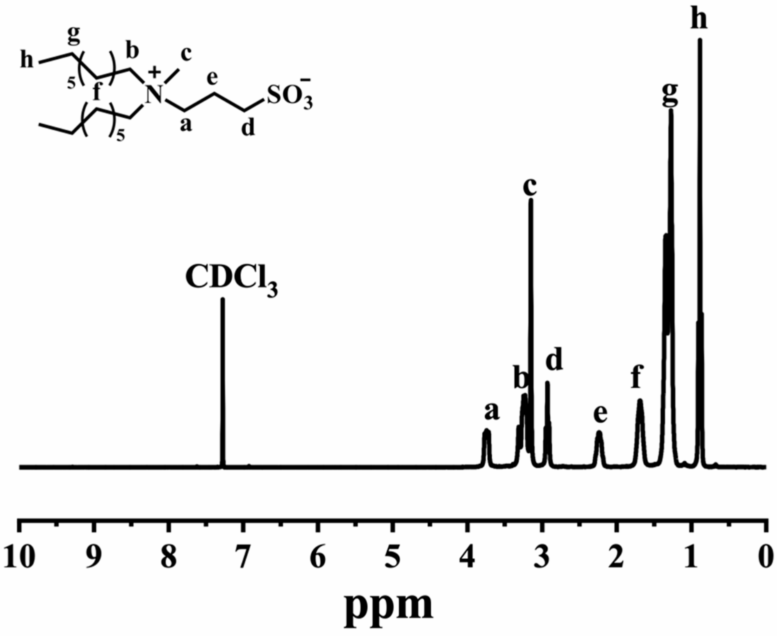

Figure S6. The ^{1}H NMR spectrum of $(C8)_2MeAS$ in $CDCl_3$.
